# Pad cultures: An ethnography of continence care and its consequences for people living with dementia during a hospital admission

**DOI:** 10.1177/14713012221116490

**Published:** 2022-07-21

**Authors:** Andy Northcott, Paula Boddington, Katie Featherstone

**Affiliations:** Geller Institute of Ageing and Memory, 7364 University of West London, Ealing, London, UK

**Keywords:** dementia, continence, incontinence, acute care, hospital, ethnography, qualitative

## Abstract

**Background:**

There is little research examining how continence care is organised and delivered to people living with dementia across an acute hospital admission, despite the prevalence of this patient population and their vulnerability within these settings.

**Objective:**

To explore how continence care is delivered to people living with dementia during an acute hospital admission.

**Design:**

Ethnographic.

**Setting(s):**

Acute medical units and wards within three hospitals across England and Wales.

**Participants:**

People living with dementia and ward staff (registered nurses and care assistants) on participating wards.

**Methods:**

Ethnographic fieldwork collected over a period of 12 months (180 days of non-participant observation) focussing on the organisation and delivery of continence care to people living with dementia. Observations were supported with in situ ethnographic interviews (*n* = 562) with patients, visitors and staff within the six observed wards. Data collection and analysis drew on the theoretical sampling and constant comparison techniques of grounded theory.

**Results:**

The findings comprised of five overall themes: (1) visibility of continence; (2) rationales of continence care; (3) containment and contagion; (4) consequences of continence care and (5) supporting continence.

**Conclusions:**

We introduce the term ‘pad cultures’ to refer to the established routine use of continence pads in the care of a wider group of people living with dementia (regardless of continence status and independence), with the rationale to provide safeguards, ensure containment and prevent ‘accidents’ or incontinent episodes. There was an expectation within acute wards that people living with dementia not only wear continence pads but that they also use them.

## Introduction

The acute hospital ward is a key site of care for people living with dementia. People living with dementia are one of the largest inpatient populations, with estimates ranging between 20% and 50% of admissions to acute beds in England and Wales (pre-Covid) (Alzheimer’s Society, 2016; Royal College of Psychiatrists, 2013), which reflects the prevalence estimates for people living with dementia within acute hospitals internationally, which range between 12.9% and 63.0% across studies (Mukadam & Sampson, 2011). A diagnosis of dementia is closely associated with an increased risk of hospitalisation (Phelan et al, 2012) representing a significant proportion of emergency admissions (77%), typically with potentially preventable and treatable conditions such as pneumonia, sepsis, urinary system disorders, frailty or long bone fractures (ARUK, 2019). Thus, the prominence of the acute hospital setting and its consequences for people living with dementia cannot be ignored (Alzheimer’s Society, 2016; Care Quality Commission, 2014; Health Foundation, 2011).

Acute hospitals have been described as ‘challenging’ (Sampson et al., 2014, p. 194) and ‘dangerous’ (Mathews et al, 2013, p. 465) places for people living with dementia. Adverse events experienced by people living with dementia during an admission typically include falls, delirium, incontinence and functional decline (George et al., 2013). The associated iatrogenic impacts (Thornlow et al., 2009) of an admission include incontinence (Hofmann and Hahn, 2013), reduced mobility (Borbasi et al., 2006; Moyle et al., 2011), increased agitation (White et al., 2017), delirium (Inouye et al., 2014; Pan et al., 2018; Voyer et al., 2011), prolonged admission (Bai et al, 2014; Tan et al., 2014) and distress (De Bellis et al., 2013; Saarnio and Isola, 2009). These impacts can all result in further dependency, institutionalisation and potentially death during or following an acute admission (George et al., 2013).

Continence care for people living with dementia in acute hospital wards is of significance, identified by policymakers (Department of Health, 2006; NICE-SCIE, 2006) families and carers (Alzheimer's Society 2009; Lakey, 2009; Patients Assocation, 2009) as a key concern, associated with poor experience and outcomes for people living with dementia.

Within the acute setting, it is critical to note that the classification of a person as incontinent, or as requiring assistance with continence care during an admission, may be associated with the care environment. Rather than physical incontinence, incontinence in hospitals is functional. A person’s cognitive impairment, mobility problems, medication or the built environment of the hospital impede on the person’s ability to reach the toilet (van Houten, 2008; Yap & Tan, 2006). Containment at the bedside is a core feature of the everyday organisation and delivery of care for people living with dementia (Featherstone, Northcott & Bridges, 2019; Featherstone, Northcott, Harden, et al., 2019) and the dominant approach to the delivery of continence care for older patients in the acute setting (Condon et al., 2021; Dingwall & McLafferty, 2006; Hälleberg Nyman et al., 2017). National audits show such practices are widespread (Wagg et al., 2010). These features of the organisation and delivery of bedside care place older people and people living with dementia at high risk of developing incontinence (Furlanetto & Emond, 2016; Mecocci et al., 2005; Zisberg et al., 2011). It is estimated that between 17% (Zisberg et al., 2011) and 36% (Furlanetto & Emond, 2016) of previously continent people living with dementia will become assessed clinically as incontinent following an acute hospital admission. Hospital acquired incontinence is established as a key long-term consequence of an acute admission for people living with dementia (Lakey, 2009).

Continence care is therefore both central to the everyday organisation and delivery of care quality and crucial for the maintenance of dignity, wellbeing and quality of life for people living with dementia and older patients (Mukadam & Liningston, 2012). Incontinence is highly discrediting (Brittain & Shaw, 2007; Goffman, 1963), and combined with a diagnosis of dementia, it increases experiences of stigma, producing a powerful attack on an individual’s self and status, both during and following an admission (Bamford & Walker, 2012; Dombrowsky, 2012; Graham, et al., 2003; Lothian & Philp, 2001).

The construction of dementia is based upon the pervasive assumption that the loss of memory correlates with a loss of self (Kontos, 2005). This construction places a value on the mind over the body, assuming that a loss of the mind is ergo a loss of the self, with which the body itself becomes passive and inactive (Kontos, 2005, 2012). While there has been a trend towards examining embodiment and embodied practices (Kontos & Martin, 2013), alongside the long-standing advocacy of person-centred care (Kitwood, 1997), this paper addresses how these practices and care approaches are rarely used under the doubly stigmatised lens of both dementia (Swaffer 2014) and continence (Dombrowsky, 2012), reflecting instead the culturally established abjection and otherness of the ageing body (Gilleard & Higgs, 2011, Higgs & Gilleard, 2014). It is possible to observe, as our data will show, the embodied nature of selfhood in the independence people living with dementia have around maintaining continence during a hospital admission. It is also observable that cultures of care within hospital settings prevent and deny this act of selfhood. This denial is observable through the enforcement of the order and expectations of hospital wards and the cultures within them, not only towards continence but also the body discipline that goes with it. The assumption of the diminished self translates into a presumed lack of agency around the body, illustrated by the moral classifications that emerge around bodies that do not submit to the cultural expectations of continence within these settings.

Despite the significance of continence care in hospital settings for people living with dementia (DuBeau et al., 2009), little is known about the appropriate strategies for its organisation and delivery during an acute admission (Harari et al., 2012), with a paucity of evidence-based training and education for nursing and ward staff (Harari et al., 2012; Wagg et al., 2010). Despite the large body of work examining continence care management interventions in other care settings (Hagglund, 2010), thus far, the everyday routine organisation and delivery of continence care for people living with dementia during an acute hospital admission has received little attention. This paper addresses this, presenting an original and empirical foundation for research examining the cultures of continence care for people living with dementia and their impacts.

## Methods

This ethnographic study examines the organisation and delivery of continence care for people living with dementia within the acute ward setting. Ethnography allows us to examine the everyday routines and behaviours within and across multi-disciplinary teams (Quinlan, 2009), while also exploring the social and institutional forces of the hospital shaping the delivery of care (Greenhaigh & Swinglehurst, 2011). This paper presents our analysis, focussing on observational data examining the organisation and delivery of continence care at the bedside. This study involved the collection of a wider data set, focussed on the delivery of everyday care at the bedside, including documentary analysis and in-depth interviews and case studies, with the findings from this wider data reported elsewhere (Featherstone & Northcott, 2021, Featherstone et al., 2022).

Observational ethnographic fieldwork was carried out in six wards within three hospitals across England and Wales: three general medical wards and three Medical Assessment Units (or variants thereof), both areas of acute hospitals known to admit large numbers of people living with dementia for acute conditions.

Ethnographic data collection (ethnography includes observation and ethnographic in situ interviews) focused on staff as they delivered continence care to patients living with dementia at the bedside. Observations focussed on the everyday practices, routines and repertoires of communication around continence practices and intimate care at the bedside, patients’ expressions of the need for assistance and staff responses and rationales. In situ interviews took place amongst the ongoing activity of the wards, speaking to staff in corridors as they delivered care or recorded notes, and to patients and to their visitors at the bedside or as they moved around the ward, with responses recorded near verbatim as fieldnotes.

A variety of observational practices and strategies were utilised in the observation of these settings. Researchers stood, rarely sitting, to reflect the pace of the ward teams. Our practice involved standing in the corridors of these wards and units, from where events within them were visible while minimising the potential for obstruction or intrusion. Individual members of staff and teams were shadowed as they worked within the ward. At no point did the team directly observe continence care behind the privacy curtain or within toilets, protecting patient dignity and privacy at all times.

Data collection involved comprehensive note-taking, written up as detailed accounts. The researchers wrote extensively during periods of observation, typically carried out with the notebook in hand, while standing or walking. The fieldnotes recorded took the form of a running record of events and incidents including details and near verbatim text of conversations and interactions (including those taking place behind the curtain where the researchers had consent to do so). Note-taking was clearly visible to all within the wards (staff and patients) who had opportunities to ask questions of the researchers. Staff were granted access to examine fieldnotes if requested. Routine ward data (staffing levels and patient numbers) was also included in data collection, providing context and an understanding of the workload around both everyday care routines and continence care within these wards.

In total, *n* = 180 days of fieldwork were conducted between October 2018 and October 2019. Periods of observations varied in length from a minimum of *n* = 2 hours to a maximum of *n* = 6 hours. Interviews (*n* = 562) were short (<5 min) with topics dynamic to the activities ongoing on the ward at that time. Fieldnotes of observations and near verbatim interview text were written up into Word files (Emerson et al., 2011; van Maanen, 2011). Approximately 500,000 words of observational fieldnotes were collected, written up, transcribed, cleaned and anonymised by the researchers. Within fieldnotes, patients were referred to in the language of these wards (bed numbers and colloquialisms), and we present them as such here. While the researchers acknowledge that such language may be stigmatising and dehumanising, this also presents the cultural realities of these settings and the interactions that take place within them.

These wards and hospital sites were purposefully selected to represent a range of hospitals types, built environments, geographies and socio-economic catchments. **Site F**: a general hospital serving a largely rural population, located in a town of approximately 10k people but serving a wider population of small towns and villages, which represented both rural and post-industrial communities; **Site G:** a teaching hospital in a regional city with an urban/suburban population of approximately 500k people with significant economic inequalities and pockets of deprivation and **Site H:** a teaching hospital in a major metropolitan city serving a multicultural urban population. Access to these sites was negotiated with NHS Trusts and Health Boards, senior hospital staff, ward managers and teams in advance of planned fieldwork.

Each ward (*n* = 6) admitted between *n* = 16 and *n* = 32 patients at any one time, across bays and individual rooms. All of these wards admitted both male and female patients, typically cohorted within separate bays. Patients with a diagnosis of dementia were identified utilising a combination of ward nursing handover notes, admissions boards and signage and in discussion with the nurse in charge of the ward. The built environment of the observed wards was highly variable, ranging from a central hub with satellite bays to long corridors, sometimes with windows onto bays and rooms, other times without.

Analysis utilised the constant comparative method and theoretical sampling whereby data collection (observation and interview data) and analysis are interrelated (Corbin & Strauss, 1990; Glaser & Strauss, 1967) and were carried out concurrently (Green, 1998; Suddaby, 2006). To optimise the generalisability of our findings (Herriott & Firestone, 1983), our approach emphasised the importance of comparisons across sites (Vogt, 2002), with theoretical saturation achieved following the search for negative cases, and on exploring a diverse and wide range of data (Glaser & Strauss, 1967; Saunders et al., 2017).

Both researchers in the field were experienced ethnographers, with experience of both data collection in the acute ward setting and working with people living with dementia. Both researchers hold PhDs and work within biomedical/healthcare schools at UK Universities. They are not registered medical professionals and held no regulatory duty of care at the time of fieldwork.

The protocol for this study was designed in collaboration with people living with dementia and their care partners. Ethics Committee approval for the study was granted by the Wales Research Ethics Committee on 19 April 2018 (18/WA/0033). Approval from both Health Research Authority and Health and Care Research Wales was granted on 5 September 2018 (IRAS 239618/Protocol 4804). The research project was approved for the purposes of the Mental Capacity Act 2005, confirming that it met the requirements of Section 31 of the Act in relation to research carried out as part of this project on, or in relation to, a person who lacks capacity to consent to taking part in the project.

## Findings

The findings of this study comprised of five overall themes: visibility of continence; rationales of continence care; containment and contagion; consequences of continence care and supporting continence.

### Visibility of continence

Continence care was essential and very visible care within these acute wards. While each of the wards we observed differed significantly in design and built environment, they all share a key characteristic of a central corridor or hub, where a nurses’ station, clerk and pharmacist can be found, from which multi-patient bays and individual side rooms can be heard and observed. Bays and side rooms would either have a toilet for patients within the bay or side room (one per bay or one per room) or be close by in the corridor, for example, located directly opposite each bay entrance. More contemporary wards had more toilets, placed at intervals along corridors. What was more visible throughout these wards were not the toilets, discrete doors often out of sight of the main corridor, but rather the continence products across these wards. Continence pads were by far the most common continence product on these wards and were highly visible to all entering these wards.

The sheer volume of continence products required within each ward made their visibility to some extent inevitable, but also meant they had become ubiquitous and taken-for-granted. Boxes of disposable continence products were evident in store and supply rooms, but also stacked along corridors, at nurses’ stations (the main station, but also the satellite small desks stationed at each bay), with clearly labelled packets, open, unpacked and stacked on the mobile equipment trolleys attached to bays and side rooms and piled on bedside cabinets. They were also highly visible on the patient’s body:

The woman in bed 15 gets up to go to the toilet opposite the foot of her bed. All of the women on this bay have a record of cognitive impairment, dementia or queried dementia in the handover notes today. A large nappy-style continence pad is clearly visible under her backless hospital gown as she walks to the toilet [Site H Day 17].

Within these wards, these disposable continence products were only ever referred to as ‘pads’, without their purpose or function explained to the person. For staff, the use of pads in the routine care of people living with dementia admitted to these wards was such a cultural norm as to not require further explanation. At the bedside, staff used a restricted repertoire of repeated phrases to explain this routine feature of bedside care to the person: ‘We are just going to put you in a pad [*shouting*]’ [Site F Day 1], ‘We just need to check your pad’ [Site G Day 3], and ‘We need to change your pad’ [Site H Day 12]. Such assumed familiarity with the requirement of pads for people living with dementia was additionally problematic because for many of these patients, this could be the first time they had been placed in and were wearing a pad.

### Rationales of continence care

#### ‘Just in Case’

This widespread and everyday use of pads in the care of people living with dementia was often explained and rationalised by ward staff as a precautionary strategy, used just in case, as a safeguard for all, including for patients recognised as continent, independently mobile or self-caring. Of course, many people living with dementia within these wards did have continence and mobility issues. However, we found the widespread use of pads was not limited to those with identified continence issues associated with their dementia or their admitting condition.

The woman in bed 2 is eating chocolate from a large box on her tray table. I speak to the care assistant about her care, she tells me that she has put a pad on her ‘just in case’, but confirms the woman is continent… the care assistant talks about how lovely she is for her age, ‘she does seem with it’, despite her admission and dementia, and ‘she knows where she is’ [Site F Day 25].

This precautionary ‘just in case’ approach to continence care for people living with dementia was deeply embedded and pervasive in all of the wards observed within this study.

#### Deprioritising continence

This use of pads as a precautionary strategy had real and significant consequences for people living with dementia. Once adopted as a ‘just in case’ strategy, the routine use of pads in the care of people living with dementia resulted in the maintenance of continence being deprioritised, and the precautionary strategy became an expectation that patients living with dementia not only wear pads but that they could and should use the pad. We found this expectation was a feature of all these ward cultures, regardless of an individual’s continence, independence or preference.

Of course, many people living with dementia (and older people) did have episodes of incontinence or were incontinent, and indeed, we observed many people call for assistance too late, after they had used their continence pad:

The nurse goes over to bed 10 and asked if he's okay. and if he would like to have his pad changed. He says that he has needed it changed for a while, and the nurse tells him that he has to tell her these things and that when she asked before he said it was all right [Site H Day 4].

However, we found that the use of pads as standard care for people living with dementia contributed to expectations that patients lacked independence, mobility and continence.

The communication of a continence need, and a request to go to the toilet by a person living with dementia, was often answered by ward staff with the commonly used phrase, ‘You’ve got a pad on’, indicating to the patient they should remain in bed and to use the pad. Here, a nurse (who is covering the bay during staff breaks and tells me she does not know these patients) supports a woman living with dementia to walk to the bathroom. It is clear that this woman is both mobile and continent, although she is lacking confidence in walking without support to the bathroom. However, as this example shows, supporting a patient to the bathroom was unusual here and can be questioned.

After being told to stay in her chair, the woman in bed 17 asks the nurse from bay B, who is covering for the regular nurse’s break, if she can go to the toilet. 17 is really appreciative of the nurse for helping her with this, ‘Thank you. Oh, you have a little room.’ They open the toilet door and it is as if she has not seen this toilet before, even though it is located within the bay. ‘Is there a light?’ The nurse turns the lights on for her, ‘Oh, thank you. Thank you so much’. The nurse explains to her that ‘When you are finished, pull the red one and I'll come back,’ pointing out the buzzer. 17 doesn't seem to understand this. She responds, ‘Will you stay here and help me back, I'm almost finished.’ ‘Okay,’ says the nurse and stays in the toilet with her. 17 finishes and they walk back to her bedside. As the nurse turns to leave, the other nurse returns from her break, so she lets her know 17 has just been to the toilet. The returning nurse responds by questioning this, ‘Why? She's got a pad on’ [Site H Day 11].

Later in the day, the same woman clearly states, ‘I need the toilet’. This time she is met with a very different response; this is clearly bewildering for the person and leads to high levels of distress for both the person and the team caring for her.

The woman in bed 17 gets up and moves to her bedside chair but remains standing up. She announces, ‘I need the toilet’, to which the response of the one-to-one health care assistant (who is closely monitoring this patient and the person in the bed next to her, bed 16) is to remind her that she has a pad on. This woman (bed 17) responds to this by reaching down and beginning to take the pad off. The one-to-one health care assistant tells her to pull it back up and again reminds her, ‘You have a pad on, you can just go there’. She (the woman in bed 17) appears to be confused by this and she again tries to take the pad off and sits on the bedside chair. The other team member in the bay suddenly shouts across the bay, ‘Wait a minute, that's not the toilet! Wait a minute!’ She (the woman in bed 17) looks confused and says, ‘I can't wait I need to go… I'm going to do it here’. The member of staff now keeps asking her to ‘sit down’ [Site H Day 11].

#### ‘Falling Behind’

Within these wards, there was a palpable tension amongst nursing and care staff of the fear of ‘falling behind’ during a shift. This relates to internal factors, such as completing the bedside observations and medicine rounds or other routine timetabled care to meet the wider organisational requirements, such as completing routines prior to the medical team rounds or deliveries of meals from the catering service.

Continence care was often deprioritised in order to meet these organisational pressures and timetables during shifts. Pads created an organisational environment where an individual’s continence needs and the act of toileting could be delayed, contained at the bedside, while other routines of bedside care perceived by ward teams as more time-critical were prioritised, such as medications in the example below:

The nurse in charge is talking to the nurse on bay 5, checking she is okay. The nurse responded to her, detailing her ‘coping mechanism’: they are going to get all the crucial medicine rounds done, then afterwards, manage as they can with tasks that are less critical [Site H Day 26].

While a patient will often have urgent needs relating to their continence, staff often judged they did not have the time and resources to immediately address this urgent care need. Here, this care assistant continues with other routines (this includes recording care and changing a bed) within the bay, deprioritising a pressing continence need with significant impacts on the person’s dignity and comfort, until the routines of the ward can accommodate continence care:

15:57 22’s visitor comes out and speaks to the other care assistant at the nursing station. He explains to her that his dad has ‘had a poo’ and has spread it all over himself and his bed, ‘he has poo all over his hands’. The care assistant acknowledges this but doesn’t get up, instead she spends a few minutes finishing what she is doing, typing on a computer at the nurses station. She then gets up, puts on a pair of blue latex gloves in the corridor, and then goes to another bay, going behind the curtain with the patient in bed 15.

16:10 Staff come back from their breaks. The care assistant leaves the bay laden with dirty laundry. 22’s son is still pacing the corridor, watching the nurses chat and do other jobs while his dad waits in the bed for help. The care assistant comes back and sits down at the station next to the returning nurse, saying to her, ’22 needs to be changed, can’t do it myself’ [Site H Day 30].

The above exchange in many ways highlights the disconnect between patient and staff around the value and significance of continence care. For staff, it was viewed as an everyday, time-consuming, part of the routine care work within every shift. Managing ‘wet’ and ‘dirty’ bodies and continence products is everyday routine work for ward staff, despite being unfamiliar and distressing for people living with dementia and their families.

### Containment and contagion

#### Permissions and containment

An impact of the widespread use of pads to manage continence care was the reinforcement of the need for people living with dementia to remain in bed or at the bedside and the requirement to seek permission to walk to the toilet. The use of pads meant staff perceived this eliminated the need for the patient to leave the bed, or indeed to interact with staff outside of the timetabled routines of continence care. With continence contained, other crucial aspects of care for the person living with dementia, such as food, medication and observations, could also be contained and delivered at the bedside. This meant large numbers of people living with dementia remained immobile, inert, apathetic and sleepy, contained in bed throughout their admission.

It is so quiet, but even when there are empty beds and little to do, the patients admitted with dementia are dressed in continence pads and just left unsupervised in their beds, with no interaction from staff. I have seen no prompting to go to the toilet. Fewer patients and fewer tasks do not seem translate to more engagement or more care, just an easier day. The team are either writing up notes or are not visible [Site H Day 24].

It was common to find people living with dementia in a state of inertia, confined to their beds in pads, at the beginning of each period of observation on these wards and still there at the end, without ever moving.

Only one patient has been out of bed to go to the toilet across all 4 bays in the last two and a half hours [Site H Day 22].

In turn, this containment made their needs, continence and otherwise, less visible to staff within the ward. The patients were perceived as quiet, so to prompt them for care would be to disturb them.

Pads transform continence, replacing active support to enable independence with timetabled routines contained and limited to the bedside: ‘pad checks’, pad changes and cleaning rounds, tasks that can be carried out in ritualistic order bed by bed within a bay of four to six beds, much like the medication or observation rounds of care. Tasks such as pad checks were typically carried out by care assistants, with continence deprioritised to a timetabled privilege, first requiring permission from the nurse or care assistant.

However, the permissions required from patients were often simply presumed. Staff would talk about and physically inspect patients, often intimately, by informing the person they were ‘checking your pad’. Requests to do so were almost always rhetorical and fitted an organisational timetabled requirement. Pad checks could involve checking for the smell of urine or faeces on the person, a physical examination using touch or using sight to check if a person’s pad had been used. Beyond euphemisms including ‘wet’ and ‘dirty’ and nouns like ‘pad’, the intrusiveness of these approaches was not considered, nor the embarrassment faced by a person disclosing they have soiled themselves and need help.

‘Shall we stand you? We came to check and make sure you are clean.’ They draw the curtains. ‘Stand up for us [first name], we are going to check your bottom.’ They discuss her as they get gloves and a fresh pad: ‘Will she be wet?’ And they head back behind the screen, saying to her, ‘Sorry darling you don’t like it.’ ‘She was soiled yesterday, I think they are giving her laxatives, we will probably have to change her.’… The care assistant heads out taking a large folded pad out to the sluice [Site F Day 21].

#### Contagion

This use of pads and the expectations of containment, coupled with the previously discussed rationales around precaution, led to contagion, informing the care of a wider patient population. Firstly, the use of pads becomes so widespread that all patients living with dementia were treated as if they are incontinent, regardless of diagnosis, and in need of assistance from incontinence technologies even when their continence is demonstrated:

The woman in bed 6 is continent but the care assistant says she is still placed in pads. The nurse interjects and says she isn’t, and the care assistant says she just now changed her pad in the toilet, and has put it on as ‘a safeguard’. The nurse criticizes this, asking the care assistant, ‘What’s the point?’ [Site F Day 20]

Secondly, these expectations around containment, enabled by the pad as a precautionary measure, spread to include a wider population of people living with dementia and older patients within these wards, who were also placed in pads, despite both their continence, independence and their capacity:

‘We use them with the confused patients, the wrap around nappies. But they shouldn’t use them. One woman came in confused and she woke up and found herself in a wrap-around nappy, she was very upset to find she had been put in this’ [Site F Day 1].

### Consequences of continence care

These cultures of continence care have consequences for both patients and ward staff. The reliance on pads and containment at the bedside create new work, which was often labour intensive and time-consuming for staff, and routinely created significant patient distress and conflict between patients and ward staff within shifts.

On bay 2 the patient in bed 14 is hidden behind the curtains. The nurse is at the bedside for what appears to be personal care, including washing and changing clothes and sheets. She admonishes this woman, ‘Less of that!’, who grumbles as the nurse continues, ‘We need to change you… your bed smells of urine… it doesn’t look good…’ [Site H Day 5]

Staff acknowledge this distress and conflict in their discussions, recognising the requirements of remaining at the bedside and the intimate care changing pads involves, was also associated with ‘aggression’ from patients living with dementia:

The care assistant on A says, ‘After he had been to the toilet he was totally pleasant for the rest of his admission.’ The other nurses and care assistants agree, ‘The patients often switch, go from aggression to calm over admission’, saying, ‘With men it’s often because they want to go to the toilet’ [Site F Day 9].

These patterns of conflict were particularly noticeable when a person living with dementia was continent and mobile yet required a pad to be changed, especially given a reluctance amongst staff to discuss with the person what the pad is for:

On bay 3 there is shouting as a patient is having her pad changed behind the curtain… the care assistant says to the patient, ‘It’s okay, we will give you a clean one, one that’s not dirty, we’ll get you a clean one… Come on, you cannot walk around naked, there are men in the next room, there we go, okay’ [Site H Day 27].

### Supporting continence

Throughout our observations, there were also ward staff and care practices during shifts which indicated that while the use of pads dominated practice within these acute wards, there were also other approaches to continence care, with benefits for people living with dementia and the staff caring for them.

This was particularly notable within site F, where the nurse in charge emphasised continence care focused on the individual, yet still described the entrenched and everyday use of pads on older patients on her ward as ‘a bugbear’ [Site F Day 1]. Examples included all ward staff (including the matron) assisting the majority of people living with dementia (and older patients) in walking to the toilet (this also involved the use of ‘stedys’ and frames, etc.) as a priority for the morning shift. Other staff recognised this approach as anomalous with other wards:

Chat with the porter in the staff room, he is taking a break. Discuss the different wards, he also notes that the unit is a caring ward, says it’s clear and comes from the matrons, keen on caring for patients not managing them… He says other wards can be very different, some have draconian rules with bullying matrons, on both patients and staff, creates paranoia. He jokes that one matron upstairs is nicknamed Pol Pot [Site F Day 28].

This variation in ward leadership was noticeable during observations of this single ward. When the nurse in charge described above was not on duty during or on a given shift, the reliance on pads and containment would quickly return within the ward, meaning patients received variable care (and associated expectations) throughout an admission.

Such individualised continence care which supported independence and providing up to 18 patients the opportunity to go to the bathroom took time, often taking the entire nursing and care team the first hour of the morning shift to complete. Our observations identified that this had longer term benefits during these shifts, the patients within these bays were noticeably calmer, there was less distress amongst patients living with dementia, which in turn led to a calmer environment for all.

Within other wards, as seen in theme one and three, there were always individual staff members who challenged the established routine use of pads to contain continence. One effect of these approaches was that it enabled them to complete routine tasks, challenging the view discussed in the previous theme that supporting independence created additional work. In the example below, a care assistant uses the time a patient is in the bathroom to ‘turn over’ their bed, a task which often took multiple members of staff significant time when a patient and their continence care was contained within the bed. Since continence pads fitted poorly and were only designed to contain ‘accidents’ or leaks and failed to accommodate large volumes of urine or fecal waste, their use would often necessitate intimate personal care and changing clothes as well as sheets:

The patient in bed 4 is up, wearing his own pyjamas and blue dressing gown. They discuss whether he wants his own pyjamas back after a wash (he does) or some clean hospital ones. He goes into the toilet (the care assistant quickly clears a path for him) and explains the red button to him. As soon as the door is locked she dashes out, grabs a laundry basket, strips his bed, and gets it remade, all in less than a minute [Site G Day 16].

Beyond workload and timetabled care, we also saw how staff who challenged cultures of containment at the bedside could also prioritise time for significant interactions with the person, building positive carer–patient relationships that continued through shifts and admissions. In the example below, a person living with dementia and a care assistant find the short walk from a toilet placed along a corridor back to the bay an opportunity to interact and talk in a way that does not occur when a patient is contained at the bedside:

20 comes out of the toilet smiling. As they walk down the corridor, the one-to-one carer starts to dance next to him. He notices this and she starts to joke saying ‘Shake, shake, shake’ as she dances next to him. They start to talk about dancing. He used to dance. She starts asking what type of dancing he did, asking if it was classical dancing. He says, ‘Quickstep’ but she doesn't know what this is. Because he is hard of hearing and she doesn't know what these dances are, this becomes a slightly confused conversation. They're both talking about dancing. He's talking about Quickstep and Foxtrot. And she's talking about hip hop and R&B and he's never heard of these. It's a funny conversation, they are both smiling and laughing at each other, even when neither understands what the other is talking about. They stop to talk in the corridor. He asks what day it is. He's worried it's Saturday and he's going to miss the football. He supports [*local team*] - ‘till the end’ he adds [Site H Day 18].

## Discussion

We suggest that the term ‘pad cultures’ captures the range of findings from this ethnography, which together inform the continence care of people living with dementia within these acute wards. We introduce the term ‘pad cultures’ to refer to the routine use of continence pads in the care of a wider group of people living with dementia (regardless of continence status and independence) as a precautionary strategy, with the rationale to provide safeguards, ensure containment and prevent ‘accidents’ or incontinent episodes, with an expectation that people living with dementia not only wear pads but that they could and should use the pad.

The design of this study allowed us to examine how these pad cultures have become, and continue to be, an established part of the organisation and delivery of care, not only within individual wards but also across hospital settings. Our purposive sampling of hospitals and wards reflected a mix of socio-economic and cultural settings, and a variety of built environments, which enabled us to demonstrate how these pad cultures were dominant regardless. Built environment factors, such as the number of toilets and their proximity to beds, the number of side rooms or the distance to sluice rooms, did not appear as variables to these dominant cultures within our observations. Rather, previously observed dominant nursing cultures of containment, routine task based care and risk aversion, led by institutional pressures on quantifiable care delivery (Featherstone & Northcott, 2021), were also observable for continence and toilet access.

When staff discussed the pad cultures identified within these wards, they emphasised the routine use of continence pads as a precautionary ‘just in case’ strategy, preventing and containing incontinent episodes at the bedside, regardless of patient independence, mobility, capacity and functionality. The impacts on the person living with dementia were often mentioned in these discussions, such as the impacts on mobility and deterioration. However, staff also described their powerlessness and we did not see these discussions have any impacts on ward practices. Pad cultures had transformed the use of pads from a useful workaround to dominant practice, viewed as both an acceptable and mandated means of managing bedside care and a requirement if the team were to meet the organisational expectations of these institutions.

Pad cultures also responded to and reinforced wider cultures of containment and permissions, reflecting concerns about risk management; people living with dementia were expected to remain in bed or at the bedside and were required to request permission to walk to the toilet. Such practices in the long-term care of older people are recognised as coercive (Ostaszkiewicz, 2017, 2018), framing the verbal and physical act of forcing a person to accept continence pads or other methods of continence containment as abusive. Within these acute ward settings, such concerns were rendered invisible by the organisational priorities of the ward and the dominance of pad cultures that these priorities establish and enforce.

The rationales behind the staff practices of containment in continence care, which enabled these pad cultures to establish, are recognisable. The pace and timetables of bedside care within these wards are also critical here. Expectations on staff to maintain the daily organisational routines of bedside care were significant. Such institutional pressures create a palpably felt sense of anxiety amongst ward staff; the fear of falling behind with the day’s work during a shift. The development of these pad cultures represents a solution to complete the perceived, expected and frequently unmanageable pace of work, overriding the urgent needs of individual patients, in particular those admitted with a diagnosis of or with suspected dementia. The use of pads as a precautionary and containment strategy was not only a response to a person’s incontinence or their inability to independently walk to a toilet within these wards but was also believed to be required for a wider group of patients, necessitated by organisational constraints.

Organisational constraints place tangible pressures on wards, which supportive continence care could jeopardise. Instead, staff deprioritised or even ignored verbal requests and physical cues from continent patients to delay or avoid continence care to concentrate on prioritised tasks (Edwards et al., 2021). The needs of the ward are prioritised at the expense of personhood, dignity and independence for the person living with dementia. Our findings reveal pads as the facilitating technology, with pad cultures dominating in place of independent continence care.

These pad-reliant approaches to workload management have clear consequences for people living with dementia, with expediency and efficiency prioritised over personhood and dignity. These pads, and their containment of both movement and waste, reduced the need for nurses to interact with patients, they reduced opportunity to recognise embodied signs of distress or urgency, and they placed at risk the functionality and independence of the person living with dementia both during and following an admission.

While there appeared to be a disconnect in the recognition of pads, as something foreign and infantilising to patients, yet something every day and essential to staff, nurses and ward staff did recognise the impacts that the cultural dominance of pads had on people living with dementia. In particular, although nurses were aware of the potential for rapid deconditioning during an admission, many did not seem to recognise other ways of working as possible. This rationale was directly expressed to the researchers in the field and also by staff in their justifications to each other.

One result of pad cultures is that the distress and challenges to dignity of continence needs are frequently not recognised. The urgency of the person living with dementia unable to reach a bathroom, or discomfort in using a pad or wearing a wet or soiled pad, and the distress thus manifest, are all contained at the bedside. Pad cultures also create interactions with patients that are far more personally invasive than if the person had been supported to the toilet (the undressing and exposure of the body and the cleaning of genitalia, clothing and bedding), invasiveness which routinely created significant distress in the person and occurs in the relatively public space of a shared hospital bay, hidden only by a thin curtain or screen. The distress caused by such discomfort, exposure and intimate care could also become viewed by staff as a feature of a person’s dementia, requiring further supervision such as one-to-one care, reinforcing their containment at the bedside and the further diminishment of the person and their autonomy.

Throughout our analysis, it was possible to observe care which challenged these pad cultures: the individual nurse who walked patients living with dementia to the toilet and the ward sister who encouraged her staff to offer assistance to patients, but we also observed the ways in which pad cultures still dominated and dictated care. Other staff would question care that supported independence, highlighting the function of the pad. Ward leadership would also be questioned, with senior staff within the institution questioning why patients were out of bed, or why patients were being transferred from the ward without a pad.

The implications for policy and practice are clear. This paper aims to shine a light on these phenomena, revealing pad cultures and their consequences and providing a foundation for improvement, and this evidence base must be used to address these cultures and the systemic inequalities they produce. In both policy and practice, this is a human rights issue that must be addressed. However, any intervention must recognise that the pressures on nursing and care staff are tangibly felt, so any challenge to these cultures must promote practice that is both feasible and empowering for both people living with dementia and for ward staff. The wider research team will soon begin an implementation project, based on the findings of this study, collaborating with ward staff within pilot wards across a number of hospitals, and delivering evidence-based training, grounded in the realities of the everyday organisation and delivery of care and the institutional constraints of ward life, to support ward staff and to start the process to challenge these cultures of care.

## Conclusions

Continence care for people living with dementia admitted to acute hospital wards is dominated by what we term ‘pad cultures’, the everyday use of continence pads for patients living with dementia regardless of their continence status. While the organisational rationales for these approaches were clear, their consequences for people living with dementia, for staff, and for a wider population of older patients within these acute wards were significant. Our data suggests that independent and supportive continence care can have benefits for both people living with dementia and for ward staff. Future research must explore how these established cultures and practices of the routine use of pads can be challenged to enable ward staff to provide supportive continence care that prioritises independence and mobility. Institutionally, there is a need for a greater acknowledgement of the pervasiveness of these pad cultures, a recognition of the impacts for people living with dementia, and the significance of supportive continence care for both care experiences and outcomes.

### Limitations

The data are limited in understanding staff perceptions of how change could improve continence care and the acutely felt burden of delivering it. It may be that a formal interview study would reveal further insight. Overall, an in-depth ethnographic approach across a number of hospital settings has yielded important insights into how continence care is recognised, understood and managed by ward staff, and the consequences it has for people living with dementia. Data was collected before Covid-19 and the introduction of social distancing measures to hospitals.
